# Proteomic Profiling of Exosomes Leads to the Identification of Novel Biomarkers for Prostate Cancer

**DOI:** 10.1371/journal.pone.0082589

**Published:** 2013-12-31

**Authors:** Diederick Duijvesz, Kristin E. Burnum-Johnson, Marina A. Gritsenko, A. Marije Hoogland, Mirella S. Vredenbregt-van den Berg, Rob Willemsen, Theo Luider, Ljiljana Paša-Tolić, Guido Jenster

**Affiliations:** 1 Department of Urology, Erasmus Medical Center, Rotterdam, Netherlands; 2 Fundamental and Computational Sciences Division, Pacific Northwest National Laboratory, Richland, Washington, United States of America; 3 Department of Pathology, Erasmus Medical Center, Rotterdam, Netherlands; 4 Department of Genetics, Erasmus Medical Center, Rotterdam, Netherlands; 5 Department of Neurology, Erasmus Medical Center, Rotterdam, Netherlands; 6 Environmental Molecular Sciences Laboratory, Pacific Northwest National Laboratory, Richland, Washington, United States of America; Innsbruck Medical University, Austria

## Abstract

**Background:**

Current markers for prostate cancer, such as PSA lack specificity. Therefore, novel biomarkers are needed. Unfortunately, the complexity of body fluids often hampers biomarker discovery. An attractive alternative approach is the isolation of small vesicles, i.e. exosomes, ∼100 nm, which contain proteins that are specific to the tissue from which they are derived and therefore can be considered as treasure chests for disease-specific biomarker discovery.

**Materials and Methods:**

Exosomes were isolated from 2 immortalized primary prostate epithelial cells (PNT2C2 and RWPE-1) and 2 PCa cell lines (PC346C and VCaP) by ultracentrifugation. After tryptic digestion, proteomic analyses utilized a nanoLC coupled with an LTQ-Orbitrap operated in tandem MS (MS/MS) mode. Accurate Mass and Time (AMT) tag approach was employed for peptide identification and quantitation. Candidate biomarkers were validated by Western blotting and Immunohistochemistry.

**Results:**

Proteomic characterization resulted in the identification of 248, 233, 169, and 216 proteins by at least 2 peptides in exosomes from PNT2C2, RWPE-1, PC346C, and VCaP, respectively. Statistical analyses revealed 52 proteins differently abundant between PCa and control cells, 9 of which were more abundant in PCa. Validation by Western blotting confirmed a higher abundance of FASN, XPO1 and PDCD6IP (ALIX) in PCa exosomes.

**Conclusions:**

Identification of exosomal proteins using high performance LC-FTMS resulted in the discovery of PDCD6IP, FASN, XPO1 and ENO1 as new candidate biomarkers for prostate cancer.

## Introduction

Prostate Specific Antigen (PSA) is a clinically useful protein biomarker for diagnostics and follow-up after treatment for prostate cancer (PCa). Nevertheless, PSA-based screening was shown to have a high risk of overdiagnosis and overtreatment because it lacks specificity [1], [2]. In order to differentiate more accurately between benign prostate diseases and (different forms) of PCa, prevent unnecessary prostate biopsies, and support the urologist in recommending optimal treatment, new molecular biomarkers are urgently needed.

In the past few decades, a tremendous amount of research has been performed to find new and better biomarkers for PCa, often using state-of-the-art mass spectrometry technologies, but the discovery of novel low abundance protein has been generally hampered by the complexity of serum or urine [3]. Isolation of exosomes from body fluids represents an attractive approach to bypass these limitations and enable detection of candidate (low abundant) biomarkers.

Recent findings in the search for new biomarkers have revealed that small exosomes (50–150 nm), are present in serum and urine [4]. By isolating exosomes from body fluids it should be possible to overcome the dynamic range challenge and facilitate characterization of tissue/cancer-derived proteins that might more accurately represent cellular conditions. Therefore exosomes could be useful for determining individual tumor characteristics [5], [6].

In this study, our goal was to determine the presence and significance of exosomal proteins as novel candidate biomarkers for PCa by comparing exosomes from non-cancerous prostate cell lines to exosomes from PCa cell lines.

## Materials and Methods

### Cell culture and isolation

Two human immortalized prostate epithelial cell lines (PNT2C2 [7] and RWPE-1) and two PCa cell lines (PC346C [8] and VCaP [9]) were cultured in 10 T175 (175 cm^2^) culture flasks (Greiner Bio-One, Frickenhausen, Germany) up to 80–100% confluency. The PNT2C2 and VCaP cell line were cultured in RPMI 1640 (Lonza, Verviers, Belgium) and supplemented with 5% and 10% FCS, 500 U penicillin and 500 U streptomycin (Lonza, Verviers, Belgium). The RWPE-1 cell line (ATCC-LGR, Wesel, Germany) was cultured in Keratinocyte Serum Free Medium (Invitrogen, CA, USA) and supplemented with 5 ml Pen-Strep and a commercial kit containing Bovine Pituitary Extract (BPE, 0.05 mg/ml) and Epidermal Growth Factor (EGF, 5 ng/ml). The PC346C cell line was cultured in Dulbecco's modified Eagle's medium-Ham's F-12 medium (Lonza), supplemented with multiple additives as described by Marques [10].

After reaching 80–100% confluency, the cells were incubated with 25 ml serum free medium. After 48 h, the supernatant was collected and subjected to centrifugation steps of 400×*g* (10 min), 3000×*g* (20 min), and 10,000×*g* (30 min) to remove cellular debris. Exosomes were then pelleted at 64,000 *g* (110 min), and at 100,000 *g* (Sucrose gradient) for 1 h [11]. At least two separate exosomes isolations from each of the four cell lines were pooled. Total amount and concentration of exosomal proteins of the pooled samples was measured with a BCA-assay (Pierce, Rockford, IL, USA).

### Electron Microscopy (EM)

5 µL of exosomes were spotted onto Formvar-coated grids (200 mesh) and fixed in 2% paraformaldehyde. After fixation the exosomes were negatively stained using 4% uranylacetate. Grids were examined by a Philips CM100 electron microscope at 80 kV.

### Sample preparation for Mass Spectrometry

TFE (2,2,2-Trifluoroethanol) (Sigma-Aldrich) was added to the samples to a final concentration of 50%. The samples were sonicated in an ice-water bath (Branson 1510, Danbury, CT) for 2 minutes and then incubated at 60°C for 2 h with constant shaking (300 rpm). For protein disulfide bridge (S-S) reduction, DTT (Dithiothreitol) (Sigma-Aldrich) was added at final concentration of 2 mM, followed by sonication for 2 min. The samples were spun down and incubated at 37°C for 1 h with shaking (300 rpm). The samples were diluted, 5-fold with 50 mM ammonium bicarbonate (pH 7.8) prior to adding sequencing grade modified trypsin (Promega, Madison, WI) for protein digestion (1∶50 w/w trypsin-to-protein). The samples were shaken (300 rpm) over-night (16 h). Rapid freezing of the samples in liquid nitrogen quenched the digestion. All samples were concentrated down in Speed-Vac SC 250 Express (Thermo Savant, Holbrook, NY).

### Mass spectrometry

Proteomic measurements were performed using a nanoLC-MS at the Environmental Molecular Science Laboratory (EMSL), Richland, WA, USA. The analytical platform consisted of an on-line constant pressure (5000 psi) reversed-phase (C^18^) liquid chromatography (RPLC) system [150 µm i.d.×360 µm o.d.×65 cm capillary (Polymicro Technologies Inc., Phoenix, AZ)] coupled to an LTQ-Orbitrap mass spectrometer (Thermo Fisher Scientific, San Jose, CA) via an electrospray ionization (ESI) source manufactured in-house [12]. Briefly, full MS were acquired over *m*/*z* range of 400–2000 at resolution of 100,000, followed by data-dependent LTQ MS/MS for the top six most abundant ions in each full MS scan, using a collision energy setting of 35% and dynamic exclusion time of 60 s. An exponential HPLC gradient of ∼100 min (from 0–70% B) was used for each analysis, with mobile phases consisting of 0.1% formic acid in water (A) and 0.1% formic acid in ACN (B). Each sample was analyzed in triplicate, with approximately 5 µg of total peptide consumed (i.e., loaded on the column) in each analysis.

### Mass Spectrometry Data Analysis

The resulting MS data was analyzed using the PNNL developed Accurate Mass and Time (AMT) Tag pipeline [13]. SEQUEST software [14] was used to search tandem mass spectra against the UniProt human database (download on April 5 2011). Confidently identified peptides (Table S1 in file S1) were assembled into an exosome-specific AMT tag database. For comparative analyses, LC-MS features were matched against AMT tags for identification and relative MS-peak intensities were used to derive change in abundance. AMT tag approach facilitated quantitation of many more peptides than spectral counting alone. As long as a peptide was identified in at least one sample/analysis (by tandem MS), it could be quantified in all datasets where it was detected, even if the LC-MS feature was not abundant enough to be fragmented in that particular analysis. VIPER software [15] was used to correlate AMT tag entries (identified peptides) with LC-MS features relying on high mass measurement accuracy (MMA<2 ppm) and normalized elution time accuracy (NET∼2.5%). Consequently, each LC-MS feature matched back to a single peptide (AMT tag) thereby giving a peak intensity value (or relative abundance) for that peptide. For redundant peptide identifications in the case of a single peptide matching multiple proteins (typically protein isoforms) a representive protein was chosen; therefore, each reported peptide matches back to a single protein. No peptide identifications were made on mass alone.

For the 263 identified proteins, the Human Protein Reference Database (HPRD) and Ingenuity Pathway Analysis (IPA) were used to determine subcellular location and biological function [16].

Selection of potential protein biomarkers for prostate cancer was performed using two independent approaches. First, proteins were selected that were present in both PCa cell line derived exosomes and absent in both non-PCa exosomes. With the second approach, DAnTE software [17] was used to convert peptide peak intensity values to a log2 scale and assess them at a protein level using Rrollup (reference peptide based scaling) parameters where peptides were excluded from scaling if they were not seen in at least three datasets and no minimum peptide presence was required. Proteins presented in this manuscript were identified by at least two peptides. ANOVA pairwise comparisons between each PCa and control cell line were also performed in DAnTE where the minimum number of data points per factor level was set at three, so that in order for a protein to show statistically significant changes it would have to be identified in all three replicates. Significant difference was determined as a p-value and q-value lower than 0.05. DAnTE generates p-values and estimates their q-values. The q-value of a test measures the proportion of false positives incurred (called the false discovery rate) when that particular test is called significant. Only the significantly different proteins were selected for unsupervised hierarchical clustering. Cluster and TreeView software was used to log transform, mean center relative expression values, and subsequently hierarchical cluster all the proteins based on their expression [18]. To further select the most promising proteins, a≥1.5 log2 fold change cutoff was applied along with a requirement that each protein showed significant change in at least two of the four comparisons listed in Table 1. Table 1 lists the resulting 52 proteins, 9 of which showed increased (and 43 decreased) abundance in exosomes derived from the PCa cells.

**Table 1:** Proteins expression differences.

Protein Description (UniProt Accession #)	Gene Symbol	PC346C vs PNT2C2	PC346C vs RWPE	VCaP vs PNT2C2	VCaP vs RWPE
Programmed cell death 6-interacting protein(Q8WUM4)*	PDCD6IP	1.64	3.28	1.95	3.59
Elongation factor 1-alpha 2(Q05639)	EEF1A2	1.92	3.18		1.83
Fatty acid synthase(P49327)*	FASN	1.67	4.06		2.52
Ubiquitin-60S ribosomal protein L40(P62987)	UBA52		2.44	1.98	3.03
Vacuolar protein sorting-associated protein 28 homolog(Q9UK41)	VPS28	2.22	3.14		2.13
Actin-related protein 3B(Q9P1U1)	ACTR3B		5.71		5.27
Basal cell adhesion molecule(P50895)	BCAM			1.95	1.95
CD9 antigen(P21926)*	CD9		4.13		2.58
Polyadenylate-binding protein 1(P11940)	PABPC1	2.89		3.24	
					
14-3-3 protein beta/alpha(P31946)	YWHAB	−4.88	−4.11	−2.42	−1.64
Annexin A2(P07355)	ANXA2	−7.86	−5.10	−5.60	−2.84
Sodium/potassium-transporting ATPase subunit alpha-1(P05023)	ATP1A1	−3.23	−2.87	−3.38	−3.01
Sodium/potassium-transporting ATPase subunit beta-1(P05026)	ATP1B1	−3.68	−3.03	−3.52	−2.87
Sodium/potassium-transporting ATPase subunit beta-3(P54709)	ATP1B3	−2.79	−2.04	−2.33	−1.58
Basigin(P35613)	BSG	−2.90	−3.56	−4.62	−5.28
Chloride intracellular channel protein 1(O00299)	CLIC1	−5.03	−2.85	−4.34	−2.16
Integrin alpha-6(P23229)	ITGA6	−2.34	−4.84	−2.21	−4.72
Junctional adhesion molecule A(Q9Y624)	F11R	−1.56	−1.58	−2.15	−2.17
Actin, aortic smooth muscle(P62736)	ACTA2	−3.61		−3.97	−1.83
Potassium-transporting ATPase alpha chain 2(P54707)	ATP12A	−3.67	−2.32	−2.89	
Catenin beta-1(P35222)	CTNNB1	−5.02	−1.73	−2.90	
Alpha-enolase(P06733)*	ENO1	−3.63	−1.82	−2.18	
78 kDa glucose-regulated protein(P11021)	HSPA5	−3.86		−3.56	−2.08
Importin subunit beta-1(Q14974)	KPNB1	−5.13	−2.02	−2.15	
Pyruvate kinase isozymes M1/M2(P14618)	PKM2	−4.02	−2.88	−2.19	
Triosephosphate isomerase(P60174)	TPI1	−3.67	−2.19	−1.62	
14-3-3 protein epsilon(P62258)	YWHAE	−3.70	−2.73		
14-3-3 protein theta(P27348)	YWHAQ		−3.17		−1.85
4F2 cell-surface antigen heavy chain(P08195)	SLC3A2	−4.01	−5.90		
ADP-ribosylation factor 1(P84077)	ARF1	−3.81		−3.12	
CD151 antigen(P48509)	CD151			−4.41	−3.12
Coxsackievirus and adenovirus receptor(P78310)	CXADR	−2.70		−1.96	
EH domain-containing protein 4(Q9H223)	EHD4	−2.77	−2.18		
Prostaglandin F2 receptor negative regulator(Q9P2B2)	PTGFRN		−1.91		−2.76
Putative heat shock protein HSP 90-beta 2(Q58FF8)	HSP90AB2P	−3.60	−1.87		
Putative heat shock protein HSP 90-beta-3(Q58FF7)	HSP90AB3P	−4.05		−2.35	
Hemoglobin subunit beta(P68871)	HBB		−5.21		−5.19
Ras GTPase-activating-like protein IQGAP1(P46940)	IQGAP1	−5.14		−4.40	
Keratin, type I cytoskeletal 9(P35527)	KRT9	−1.54	−1.88		
Keratin, type II cytoskeletal 2 epidermal(P35908)	KRT2	−1.64	−1.92		
Lactadherin(Q08431)	MFGE8	−2.02	−2.19		
Protein DJ-1(Q99497)	PARK7	−1.80	−2.37		
Phosphoglycerate kinase 1(P00558)	PGK1	−2.28		−2.13	
Peroxiredoxin-1(Q06830)	PRDX1	−2.43		−2.14	
Ras-related protein Rab-10(P61026)	RAB10	−3.10		−3.11	
Ras-related protein Rab-1A(P62820)	RAB1A	−2.93	−2.11		
Ras-related C3 botulinum toxin substrate 1(P63000)	RAC1		−1.63		−2.42
Ras-related protein Rap-1A(P62834)	RAP1A	−3.01		−2.95	
Adenosylhomocysteinase(P23526)	AHCY	−1.97	−1.68		
Tubulin alpha-1A chain(Q71U36)	TUBA1A	−3.42		−1.80	
T-complex protein 1 subunit epsilon(P48643)	CCT5	−2.72	−2.85		
UDP-glucose 6-dehydrogenase(O60701)	UGDH	−3.58		−3.13	

Proteins with significant abundance changes (>1.50 log2 fold) between prostate cancer and immortalized primary prostate epithelial cell lines.

To further select the most promising proteins from the two approaches, proteins were scaled based on prostate preferentiality. Five different human gene expression atlases [19]–[23] based on microarray expression data were combined in SRS [24], to determine protein-corresponding gene expression. Eventually, prostate preferentiality was determined as 1.5 fold higher expression in prostate tissue compared to kidney and bladder tissue using gene expression microarray data [25].

### Western blotting

From every exosome sample 5 µg of protein was mixed with Laemmli sample buffer (1∶1), heated at 95°C for two minutes and loaded onto 10% one-dimensional SDS-PAGE gels. Subsequently, proteins were transferred onto Protran nitrocellulose membranes (Whatman's Hertogenbosch, the Netherlands) and blocked (1 h) at room temperature with 5% nonfat dry milk in Tris-Buffered Saline with 0.1% Tween-20. Then, the gels were incubated overnight at 4°C with antibodies against: PDCD6IP (1∶500 dilution, Sigma-Aldrich), FASN (1∶500 dilution, Sigma-Aldrich), XPO1 (1∶200 dilution, Santa Cruz Biotechnology, Heidelberg, Germany), ENO1 (Clone H300, 1∶1000 dilution, Santa Cruz Biotechnology), GAPDH (Clone 7B, 1∶500 dilution, Santa Cruz Biotechnology), CD9 (Clone 209306, 1∶500 dilution, R&D Systems, Abingdon, UK), PSA (Clone A0562, 1∶500 dilution, DakoCytomation, Heverlee, Belgium). Secondary antibodies (HRP-conjugated Goat anti Mouse/Rabbit, 1∶10,000 dilutions, DakoCytomation) were incubated for 1 h. BM Chemiluminescence Blotting Substrate (POD, Roche Applied Science, Almere) was used to initiate the oxidation by HRP.

### Immunohistochemistry (IHC)

IHC expression analysis of candidate biomarkers was performed on: normal prostate tissue (NAP, n = 2), PCa Gleason score 3+3 = 6 (n = 2), and PCa Gleason score 5+4 = 9 (n = 2). Tissues slides were mounted on aminoacetylsilane coated glass slides (Starfrost, Berlin, Germany), deparaffinised in xylene and dehydrated in ethanol. Endogenous peroxidase activity was blocked with 0.3% hydrogen peroxide in PBS for 20 min. Microwave pretreatment was performed for 15 min in tris(hydroxymethyl)aminomethane-EDTA (pH 9.0). After pretreatment, the slides were incubated with the PDCD6IP (1∶400), FASN (1∶50), and XPO1 (1∶50) antibodies, overnight at 4°C. Subsequently, the EnVision DAKO kit (DAKO, Glostrup, Denmark) was used for chromogenic visualization. After staining the slides were counterstained with hematoxylin, washed, dehydrated and mounted in malinol (Chroma-Geselschaft, Körgen, Germany).

## Results

### Isolation and characterization

**Figure 1:**
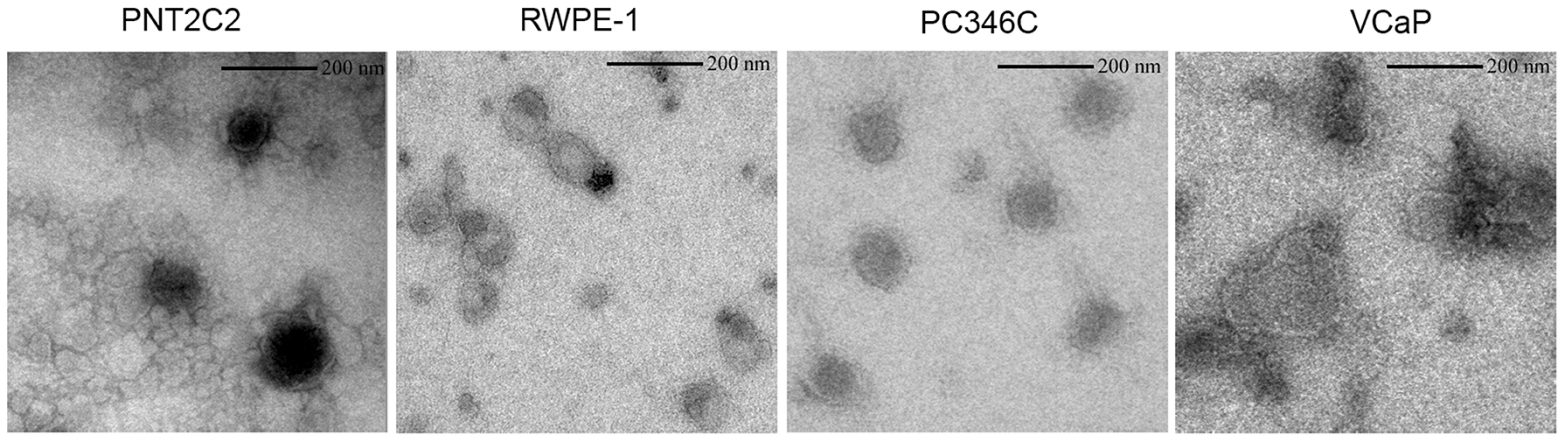
Electron microscopic (EM) images of purified exosomes derived from the PNT2C2, RWPE-1, PC346C and VCaP cell lines. All exosome samples contain multiple vesicles with a size in the range of 70–200 nm. The darkness of the vesicles reflects the difference in density of exosomes between samples.

Electron Microscopy (EM) of the purified exosome samples revealed that vesicles derived from four cell lines are reasonably homogeneous in size, with an approximate diameter of 70–200 nm (Figure 1).

**Figure 2:**
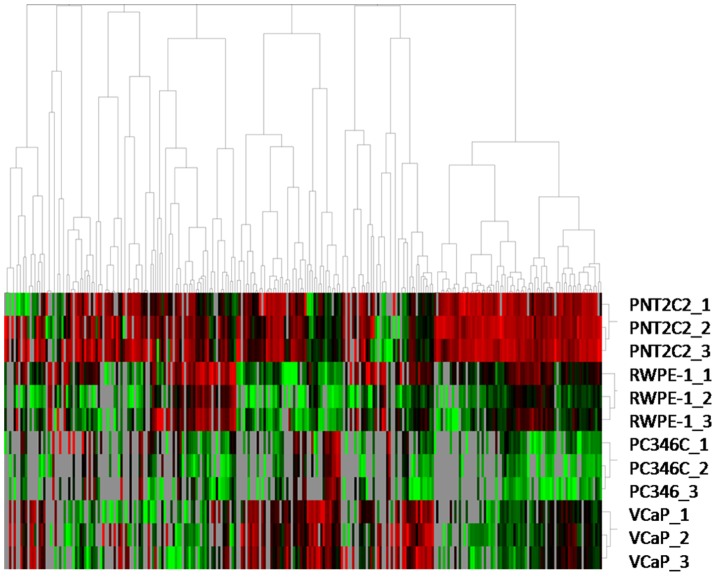
Unsupervised hierarchical clustering of differentially abundant proteins (n = 263 proteins with >2 peptides) based on their MS-peak intensity values. Each exosome sample was analyzed in triplicate. Results were mean centered and log-transformed. Relative protein abundance is colored-coded with red corresponding to a relatively high abundance, green r corresponding to a relatively low abundance, and grey indicating missing abundance values.

LC-MS/MS analyses after tryptic digestion, identified 1494 non-redundant peptides (Table S1 in file S1), corresponding to 496 proteins by at least 1 peptide (Table S2 in file S1). 263 proteins were identified by at least 2 peptides, and specifically 248, 233, 169, and 216 proteins were identified in the PNT2C2, RWPE-1, PC346C and VCaP cell lines, respectively (Table S3 in file S1). Unsupervised hierarchical clustering of these 263 proteins resulted in a clear distinction between cancer and control cell lines (Figure 2).

**Figure 3:**
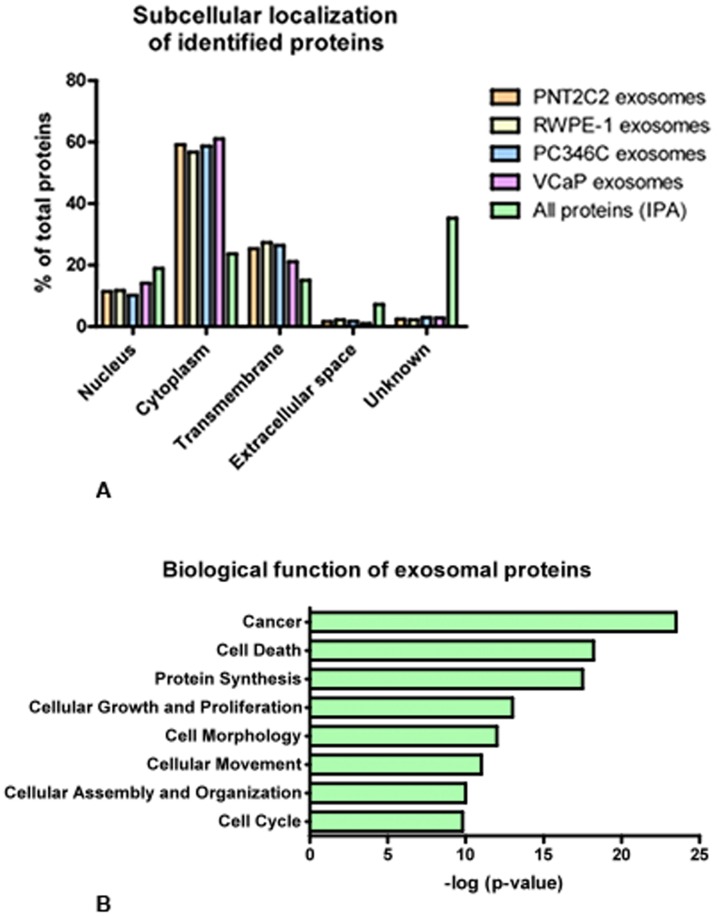
Subcellular assignment of the proteins identified within the different samples in panel A. Exosomes from all four cell lines (PNT2C2, RWPE-1, PC346C, VCaP) contained 60% of cytoplasmic proteins and 25% of transmembrane proteins. B. The top seven functions of exosomal proteins according to Ingenuity Pathway Analysis. Fisher's exact test was applied to calculate significance (p-value<0.05).

The identified exosomal proteins in the 4 cell lines showed similar subcellular localization patterns (Figure 3A). When compared to all proteins included in the IPA database, exosomes contain, relatively speaking, more cytoplasmic proteins and almost no extracellular proteins. A majority of proteins detected within exosomes relate to tumorigenesis, cell death, protein synthesis, cellular growth and proliferation (Figure 3B).

### Selection of potential biomarkers

To select proteins that show significant change in abundance between the PCa exosomes and non-PCa exosomes we used ANOVA pairwise comparisons (i.e., p-value and q-value<0.05, presence in all analyses, ≥2 peptides) [17]. Table S4–8 in file S1 contain results obtained for the PC346C (PCa) vs. PNT2C2 (control), PC346C (PCa) vs. RWPE-1 (control), VCaP (PCa) vs. PNT2C2 (control), and VCaP (PCa) vs. RWPE-1 (control). To further improve confidence, we required that each protein was determined to be significantly changing in abundance in at least 2 comparisons; this further reduced our list to 52 proteins (Table 1 and Table S9 in file S1).

Our proteomic analysis indicated PDCD6IP, FASN, CD9, and ENO1 to have significant change in abundance between two conditions, while XPO1 did not pass our stringent filtering criteria and was therefore considered unchanged in abundance in the VCaP vs. RWPE-1 comparison (Table S8 in file S1). Even so, we chose to validate XPO1 because of its higher abundance in VCaP exosomes compared to the RWPE-1 control and the availability of a high quality antibody suitable for Western blotting and immunohistochemistry.

### Exploration of novel candidate biomarkers

**Figure 4:**
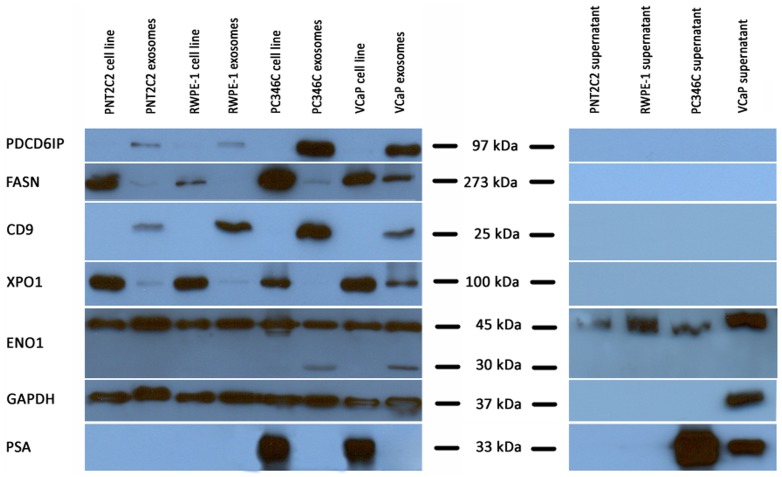
Validation of protein expression by Western blotting. All four exosome samples and their corresponding cell lines were used for validation. Furthermore, supernatant from the pelleted exosomes was used as a control. The selected proteins FASN, XPO1, CD9 and PDCD6IP, were tested with ENO1 and GAPDH as controls. PSA was tested to confirm it is secreted through alternative secretion pathway and therefore not present within exosomes. The nearest protein marker (kDa) is indicated for each blot.

For FASN and XPO1, strong signals were observed in whole cell lysates as compared to the exosomes and there appears to be relatively higher abundance within the VCaP exosome sample (Figure 4). The protein PDCD6IP is enriched in exosomes and shows higher abundance in both PCa-derived exosome samples as depicted in Figure 4 and Table 1. Based on the MS analyses, FASN is significantly higher in the PC346C exosomes compared to both controls and in VCaP exosomes compared to RWPE-1 control. This higher abundance of FASN in PC346C is confirmed by the Western blot. CD9 is highly enriched in exosomes and shows relatively high abundance in the PC346C exosomes. XPO1 exhibited higher abundance in the VCaP exosomes compared to controls. MS data characterized ENO1 to be significantly decreased in abundance in PC346C compared to both controls and in VCaP compared to the PNT2C2 control. Western blotting of ENO1 revealed an additional band (approximately 30 kDa) within the two PCa-derived exosome samples. As expected, based on the difference in PSA-secretion and exosome formation, PSA is predominately present in the two cancer cell samples and absent in exosomes. Supernatants that were collected after exosomes were pelleted during ultracentrifugation, did not contain any of the exosomal proteins, except ENO1 and GAPDH uniquely in VCaP medium.

### Verification of expression in clinical samples

**Figure 5:**
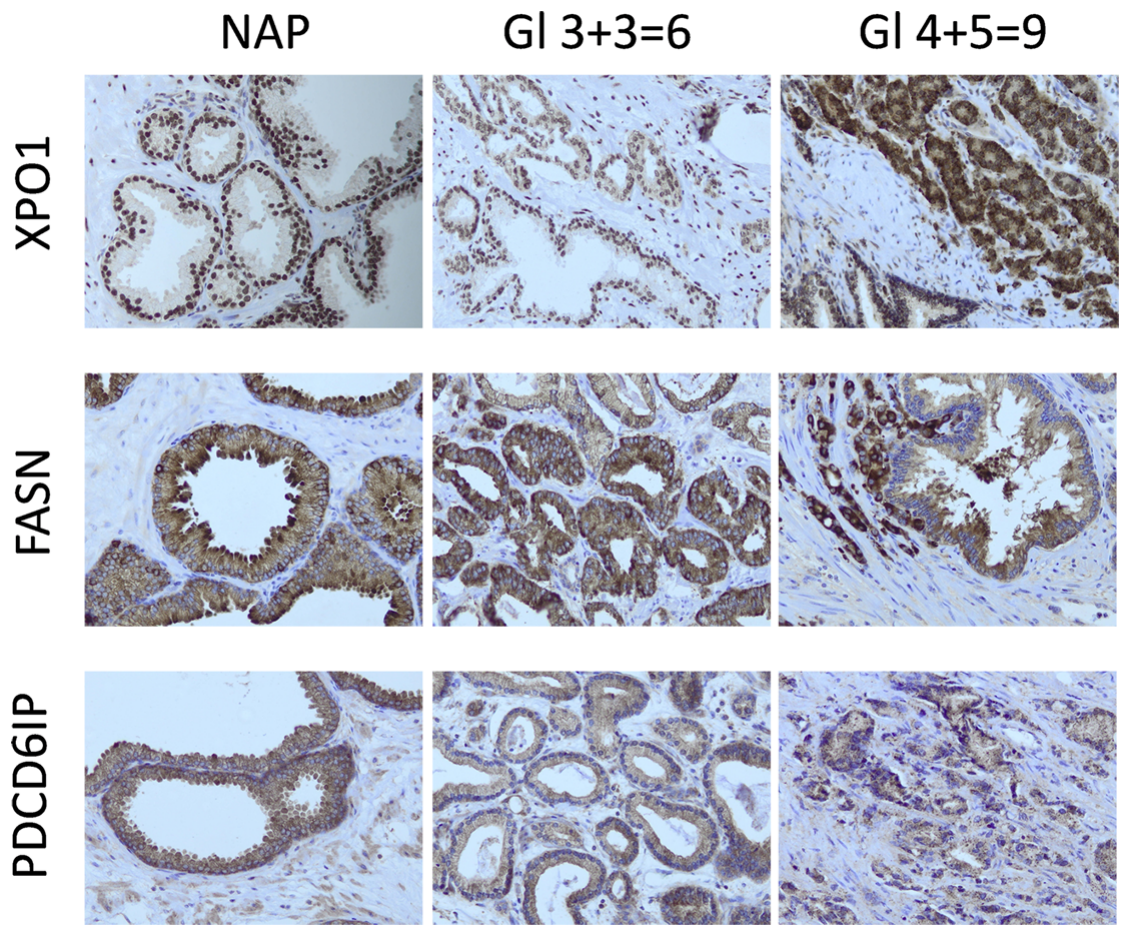
XPO1, FASN and PDCD6IP abundance by immunohistochemistry on normal adjacent prostate (NAP), low-grade prostate cancer (Gleason score 3+3 = 6) and high grade prostate cancer (Gleason score 4+5 = 9). Representative pictures of the staining from 2 independent samples per group.

PDCD6IP showed strong luminal and basal epithelial cytoplasmic staining in normal adjacent prostate (NAP), with no alteration in protein expression in PCa tissue with different Gleason scores (Figure 5). In NAP, FASN is moderately to highly abundant in epithelial cells. Nevertheless, when Gleason scores increases, staining becomes stronger. Regarding XPO1, there is a strong nuclear abundance in NAP and a weak cytoplasmic staining. Within PCa cells, the cytoplasmic abundance increases with Gleason scores. Nuclear staining remains equal among all PCa tissues.

## Discussion

Comparison of exosomes derived from cancer cell lines and immortalized prostate epithelial cells, provides a powerful tool to overcome the dynamic range challenge and identify novel low abundant cancer-derived biomarkers. This unique approach within exosomal protein research, combined with state-of-the-art LC-MS analyses facilitated identification of novel candidate biomarkers for PCa. This study describes exosomal protein expression from multiple PCa cell lines, but also examines exosome content from multiple immortalized normal prostate epithelial cells. As compared to previous studies, this enables us to more reliably identify PCa-specific candidate biomarkers and common exosomal proteins. Using Western blotting and IHC, we verify differential expression and show that the candidate markers are expressed by prostate cancer cells in patient samples.

The total number of unique proteins we identified in this study (496 by ≥1 peptide, 263 by ≥2 peptides), is comparable with previously published exosome proteomic reports. [26]–[30]. Assignment of a subcellular localization revealed that a large proportion of exosomal proteins normally locate in the cytoplasm or nucleus of cells. After comparing this to a database containing a vast majority of all proteins (∼20,000), we noticed that exosomes have a relatively comparable abundance of nuclear proteins, higher abundance of cytoplasmic proteins and a substantially lower abundance of extracellular proteins. This fits the current theory of exosome formation [4]. Exosomes display an over-representation of transmembrane and cytoplasmic proteins, such as CD9 and PDCD6IP, as shown by Western blots. This finding agrees with the theory that biogenesis and selection of exosomal content is not a random procedure, but at least partly the result of a selective sorting process [4].

**Figure 6:**
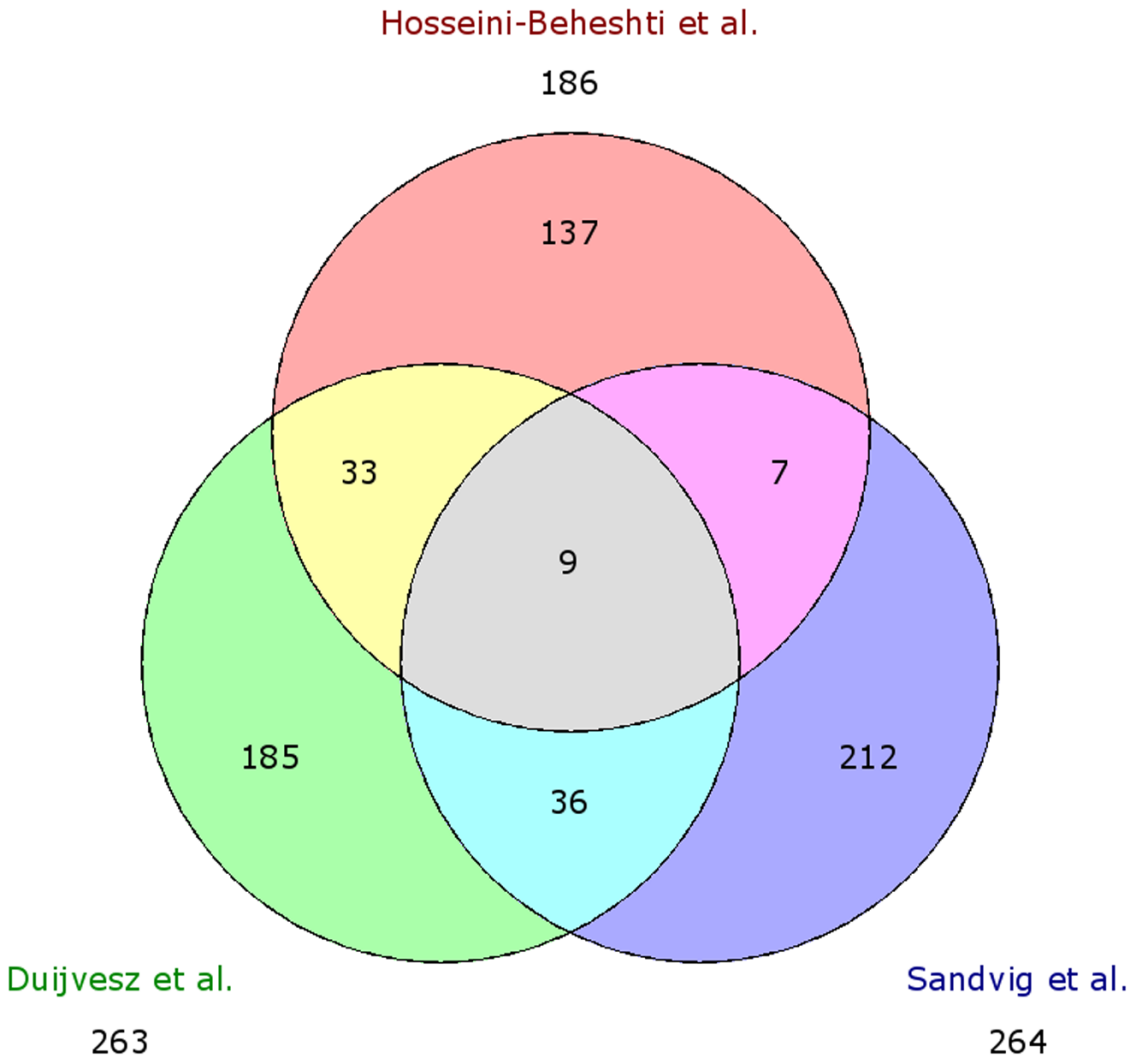
Comparison of proteins identified by Hosseini-Beheshti *et al.*, Sandvig *et al.* and this study visualized by a Venn diagram. The number of proteins identified in each study are a compilation of the cancer-derived exosomal proteins identified by MS-MS.

Two recent proteomic studies revealed exosomal proteins related to prostate cancer [30], [31]; Sandvig *et al.* performed LC-MS analysis on a single prostate (cancer) cell line, were Hosseini-Beheshti *et al.* examined five PCa cell lines and one non-malignant prostate epithelial cell line. They both reported 266 and 220 proteins, which is similar to the number we revealed. Interestingly, Sandvig *et al.* reported a different protein subcellular distribution and correlation with biological processes as compared to our data. Sandvig *et al.* proposed CDCP1 and CD151 as candidate markers, were Hosseini-Beheshti suggested ANXA2, CLSTN1, FLNC, FOLH1 and GDF15. Hosseini-Beheshti *et al.* also showed FASN to be an exosomes-derived candidate biomarker (in agreement with our results). When we compare their identified proteins (by >2 peptides) we noticed an overlap of only 9 proteins, respectively CD9, ANXA1. ACTB, PGK1, RAN, EPCAM, HSPB1, PDCD6IP and PRDX1 (Figure 6). These proteins have been published previously in multiple articles and are considered to be present in almost all exosomes. PDCD6IP has also been identified by both researchers, but has not been found to be more abundant in PCa-derived exosomes. A total of 199 proteins have uniquely been found in our study, including the candidate biomarker XPO1. The overlap between the studies is rather limited. Particularly, an overlap of only 42 proteins between our study and Hosseini-Beheshti *et al.* is not expected since party the same cell lines were utilized (VCaP and RWPE-1). This narrow overlap could be the result of different purification, MS-MS technologies and data processing pipelines utilized. In addition, if exosomes contain a large collection of different proteins, overlap between databases can be limited if only a fraction of all proteins is identified in each study. A recent report by Principe *et al.* showed the first identification of more than 900 proteins in exosomes derived from human prostate fluid from patients with low grade PCa and healthy men [32]. When we compared their unique protein expression (presence of >2 peptides), only 31 proteins overlap, including multiple annexins (ANXA1-7), peroxiredoxins (PRDX1-6), PDCD6IP and general transmembrane proteins (CD9/CD242/CD44). All these proteins are thought to be present in almost all exosomes. To what extent the limited overlap is due to actual differences between cell line-derived exosomes and vesicles from clinical samples, still needs to be established.

We identified PDCD6IP as being enriched in exosomes, especially in PCa exosomes. PDCD6IP, also known as ALIX, is a cytoplasmic protein that is known for its role in apoptosis and is shown to be involved in the pathway of selected sorting by ESCRT-complexes [33]. PDCD6IP has been used as a general marker to prove the presence of exosomes [34]. However, no association was made with a higher abundance in cancer-derived exosomes. A possible explanation for high PDCD6IP abundance in PCa-derived exosomes could be that PCa cells have an altered production of exosomes, where they are unable to regulate the sorting of exosomal content properly anymore. It is also possible that cancerous cells attempt to remove the PDCD6IP protein by exosome secretion to (partially) suppress apoptosis. To complement this theory, other non-PCa related studies have shown that overexpression of PDCD6IP correlates with cell death [35]. Using IHC, we did not find any difference in PDCD6IP abundance between normal prostate epithelium and PCa tissue.

Both FASN and XPO1 have a higher abundance in PCa exosomes derived from VCaP cells. FASN catalyzes the formation of long chain acids from acetyl-CoA, malonyl-CoA and NADPH and has already been suggested as a marker for PCa [36], [37]. Recent studies showed that FASN is primarily expressed in hormone-sensitive cells, promote cell proliferation and that the inhibition of FASN effectively and selectively kills cancer cells [36]. However, these studies were all performed *in vitro*. The VCaP cell line used herein is hormone-sensitive, which could explain the higher abundance of FASN in VCaP-derived exosomes. Cancer cells produce more FASN, likely because it promotes cell proliferation, which could lead to higher incorporation into the exosomes. In agreement with previous results [38], we also observed an increased abundance in PCa as compared to NAP.

XPO1 has been suggested as a prognostic marker for other types of cancer [39]. XPO1 is a nuclear protein known to be involved in nuclear-cytoplasmic export of signal-bearing (NES) proteins, which play a role in relevant tumor signaling pathways, such as P53, AKT1, HDAC5, the androgen receptor (AR) and the EGFR [40]–[42]. Our findings indicate that XPO1 could be a potential biomarker for PCa. When this protein is validated on whole section PCa samples with IHC, we observe a strong nuclear expression and a very weak cytoplasmic expression. Interestingly, within cancer cells, this protein seems to translocate into the cytoplasm. With increasing Gleason score, cytoplasmic XPO1 expression becomes more intense. Why this process occurs remains unclear. In a normal cell, XPO1 has to be transported from the cytoplasm back in the nucleus in order to function as a chaperone protein. If this relocation process is inhibited in cancer, cytoplasmic XPO1 will accumulate and more XPO1 might get incorporated in exosomes.

As published previously, an additional protein band (approximately 30 kDa) appears with Western blotting when using an antibody directed against ENO1 in the PC346C cell line [43]. Here we show that this band is also present in VCaP exosomes and absent in exosomes from two non-PCa cell lines. The origin of the additional band could be a non-specific antibody cross-reaction to another protein, an alternative spliced ENO1, a translated fragment or a breakdown product from the original protein. A known protein isoform called MBP-1 (c-myc promoter-binding protein-1) is produced form the ENO1 gene [44]. MBP-1 is identical in sequence to ENO1 but lacks the first 93 or 96 amino acids. With a calculated molecular mass of 36 kDa, MBP-1 is unlikely the estimated 30 kDa additional band. The observation that this additional band occurs only in both cancerous samples could indicate that it might have a relation to PCa.

The new markers we identified came from cell line-derived exosomes. Although exosomal presence and cancer tissue expression of a selected set of candidate biomarkers was confirmed using Western blotting and IHC, independent validation of the candidate markers is still needed on a large collection of samples such as patient urinary exosomes or tissue samples. This will elucidate their role as a biomarker for PCa.

In order to test presence of exosomal proteins in large cohorts of patient samples, one can resort to standard IHC of candidate markers on tissue microarrays and prostate biopsies. Alternatively, intact exosomes can be isolated from urine or plasma using precipitation, filtration or immunocapture protocols after which the content of exosomes can be measured by ELISAs, specific for the proteins of interest. ELISAs are currently being developed for detection of intact prostate-derived exosomes using capture or detection antibodies directed against known prostate-specific transmembrane proteins. Such an assay enables one to identify expression of transmembrane proteins but also estimate the number of exosomes.

### Conclusion

Prostate (cancer) cells secrete exosomes that can be used to identify novel candidate biomarkers for PCa. Identification of exosomal proteins by high performance LC-FTMS resulted in the discovery of PDCD6IP, FASN, XPO1 and ENO1 as new candidate biomarkers for PCa. In the next phase, all proposed candidate biomarkers will be evaluated on patient samples (tissue, serum or urine) to fully elucidate their potential clinical value.

## Supporting Information

File S1 **Supporting information that contains 9 supporting information files.** **Table S1 in file S1:** Confidently identified peptides (n = 1494) were assembled into an exosome-specific AMT tag database. **Table S2 in file S1:** 1494 non-redundant peptides corresponds to 496 proteins by at least 1 peptide. **Table S3 in file S1:** 263 proteins were identified by at least 2 peptides, and specifically 248, 233, 169, and 216 proteins were identified in the PNT2C2, RWPE-1, PC346C and VCaP cell lines, respectively. **Table S4 in file S1:** ANOVA pairwise comparisons were applied (i.e., p-value and q-value<0.05, presence in all analyses, ≥2 peptides) to compare protein expression between PC346C (PCa) and PNT2C2 (control). **Table S5 in file S1:** ANOVA pairwise comparisons were applied (i.e., p-value and q-value<0.05, presence in all analyses, ≥2 peptides) to compare protein expression between PC346C (PCa) and RWPE-1 (control). **Table S6 in file S1:** ANOVA pairwise comparisons were applied (i.e., p-value and q-value<0.05, presence in all analyses, ≥2 peptides) to compare protein expression between VCaP (PCa) and PNT2C2 (control). **Table S7 in file S1:** ANOVA pairwise comparisons were applied (i.e., p-value and q-value<0.05, presence in all analyses, ≥2 peptides) to compare protein expression between PC346C (PCa) and PNT2C2 (control). **Table S8 in file S1:** ANOVA pairwise comparisons were applied (i.e., p-value and q-value<0.05, presence in all analyses, ≥2 peptides) to compare protein expression between VCaP (PCa) and RWPE-1 (control). **Table S9 in file S1:** To further improve confidence, we required that each protein was determined to be significantly changing in abundance in at least 2 comparisons; this further reduced our list to 52 proteins.
(XLSX)
